# Matrix Gla Protein Binds to Fibronectin and Enhances Cell Attachment and Spreading on Fibronectin

**DOI:** 10.1155/2014/807013

**Published:** 2014-08-21

**Authors:** Satoru Ken Nishimoto, Miyako Nishimoto

**Affiliations:** ^1^Department of Microbiology, Immunology, and Biochemistry, University of Tennessee Health Science Center, Memphis, TN, USA; ^2^Department of Biomedical Engineering, University of Tennessee Health Science Center, Memphis, TN, USA; ^3^Department of Medical Education, University of Tennessee Health Science Center, Memphis, TN, USA

## Abstract

*Background*. Matrix Gla protein (MGP) is a vitamin K-dependent, extracellular matrix protein. MGP is a calcification inhibitor of arteries and cartilage. However MGP is synthesized in many tissues and is especially enriched in embryonic tissues and in cancer cells. The presence of MGP in those instances does not correlate well with the calcification inhibitory role. This study explores a potential mechanism for MGP to bind to matrix proteins and alter cell matrix interactions. *Methods*. To determine whether MGP influences cell behavior through interaction with fibronectin, we studied MGP binding to fibronectin, the effect of MGP on fibronectin mediated cell attachment and spreading and immunolocalized MGP and fibronectin. *Results*. First, MGP binds to fibronectin. The binding site for MGP is in a specific fibronectin fragment, called III1-C or anastellin. The binding site for fibronectin is in a MGP C-terminal peptide comprising amino acids 61–77. Second, MGP enhances cell attachment and cell spreading on fibronectin. MGP alone does not promote cell adhesion. Third, MGP is present in fibronectin-rich regions of tissue sections. *Conclusions*. MGP binds to fibronectin. The presence of MGP increased cell-fibronectin interactions.

## 1. Introduction

Matrix Gla protein (MGP) is an essential calcification inhibitor. However, MGP has other properties that may relate to its biological role. It is synthesized in a wide variety of tissues during embryonic life [1–5]. MGP is synthesized in embryonic kidney tubules and MGP protein and vitronectin colocalize at focal sites [6]. MGP is highly expressed in the eye [7, 8]. MGP synthesis increases as intraocular pressure is increased [9, 10]. MGP is necessary for branching morphogenesis in lung [11]. MGP is highly expressed in cancers of the ovary, testes, kidney, prostate, and glioblastomas but its function in neoplastic cells is unknown [12–15]. MGP is a migration-promoting protein for glioblastoma, suggesting that it can promote the cancer spreading [16]. MGP promotes angiogenesis and growth in xenografts of glioblastoma [17].

MGP and the MGP peptide containing amino acids 61–77 bind vitronectin [6]. Vitronectin binding suggested a mechanism to anchor MGP in the extracellular matrix where it may bind to BMPs and/or calcium to prevent calcification. MGP has at least 2 functional domains, a vitronectin extracellular matrix binding domain in the C-terminal and a calcification inhibitory domain in the N-terminal half of the protein. MGP is known to inhibit soft tissue calcification. MGP deficiency results in mineralization defects. MGP-null transgenic mice suffer mineralized arteries and cartilage. Mutations in human MGP that prevent synthesis of functional MGP suffer from Keutel syndrome, with excessive calcification of cartilage and pulmonary artery stenosis [5, 18, 19]. Excessive calcification occurs if MGP is absent, but the mechanism of calcification inhibition is unknown. A postulated mechanism of calcification inhibition is regulation of bone morphogenetic protein-2 (BMP-2), a cytokine/morphogen in the transforming growth factor beta superfamily [10, 20–23]. A role in transport of calcium-phosphate complexes has also been suggested, because MGP binds calcium phosphate crystals and forms a serum complex with fetuin-A and calcium phosphate [24, 25]. Calcification inhibition by MGP is connected to the GLA residues contained in the N-terminal half of MGP which are involved in binding of BMP-2, hydroxyapatite, and fetuin-A calcium phosphate complexes [22, 24–26]. The GLA domain includes the vitamin K-dependent, gamma-carboxyglutamic acids (GLA), and all 3 phosphoserines [2, 27, 28]. The functional role of GLA and the GLA domain is supported by calcification defects that occur after treatment with warfarin, an inhibitor of GLA formation [29, 30].

The present studies show MGP binds fibronectin and augments cell adhesion and spreading on fibronectin. MGP localizes with fibronectin in many embryonic tissues, possibly augmenting cell matrix interaction in development. One aspect of MGP function is mediated by binding fibronectin and increasing cell interactions with fibronectin.

## 2. Materials and Methods 

### 2.1. Materials

Matrix Gla protein was purified as described from bovine bone, with the final step being reversed phase purification on a C18 column [31]. After purification on HPLC, the purified MGP migrated as a single band on SDS-PAGE. MGP is a relatively insoluble protein with an approximate water solubility of 10 *μ*g/mL; purified MGP was kept at −20°C in a solution of approximately 40% acetonitrile and 0.06% trifluoroacetic acid in water. A typical purification peak contained 0.2 to 0.4 mg/mL. The final concentration of MGP was achieved by dilution into aqueous buffer at 10 *μ*g/mL or lower. Concentration of MGP was estimated by absorbance at 280 nM, using an extinction coefficient of 1.0 mg/mL = 1.0 absorbance units. Rabbit antibodies to bovine MGP and the purified IgG fraction were produced as described using Pierce Immunopure columns [6, 31]. The MGP peptide corresponding to amino acids 61–77 in the secreted human protein NH_2_-ERYAMVYGYNAAYNRYF-COOH was synthesized by the Molecular Resource Center of the University of Tennessee, HSC. Bovine serum albumin, human plasma fibronectin, and its proteolytic fragments, including the III1-C polypeptide (anastellin), guinea pig liver tissue transglutaminase (tTG), human fibrinogen, gelatin, pooled normal rabbit serum, were purchased from Sigma. Vitronectin was purchased from Fisher (BD Biosciences). 353915 Falcon 96-well elisa plates were used for cell attachment assays. Lab-Tek II glass 4-chamber slides were purchased from Nalge Nunc. Reagents for immunohistochemistry were from Vector Labs. The reducible crosslinking chemical Dithiobis (succinimidyl proprionate) (DSP) and the Immunopure IgG Purification Kit were purchased from Pierce. Electrophoresis and blotting chemicals were from Bio-Rad Laboratories unless otherwise indicated. All chemical components of buffers were analytical reagent grade or better.

### 2.2. Solid Phase Protein Binding Assay

Protein binding was assessed by the ability of filter immobilized target proteins to bind to iodinated MGP in an overlay buffer. Purified MGP to use as a tracer is iodinated as described previously [31]. The methods for assessing MGP binding by nitrocellulose transferred proteins from SDS polyacrylamide gels (SDS-PAGE) and electroblotting were described previously [6, 31–33]. Briefly, proteins were run in an SDS-PAGE and then transferred to Shleicher and Schuell BAS-NC reinforced nitrocellulose membranes by electroblotting on a Hoefer electroblot apparatus [6]. In some experiments proteins were transferred to membranes by dot-blotting on a Bio-Rad Bio-dot apparatus as described in [6]. The membranes are incubated in 1% bovine serum albumin (BSA), 10 mM phosphate, 140 mM NaCl, and pH 7.4 (BSA-PBS) overnight at 4°C. The filter overlay is performed in BSA-PBS with 0.1% tween 20 buffers containing radiolabeled MGP for 4 hours at ambient temperature and then washed in incubation buffer. The membrane is exposed to Kodak Biomax MS film in a Kodak Biomax Transcreen-HE Intensifying Screen and developed on an automatic film developer. An AlphaImager 2000 Documentation and Analysis System with digital camera acquired images.

### 2.3. Crosslinking of MGP with Proteins

Fibronectin (FN), fibrinogen (FG), and vitronectin (VN) were mixed with tissue transglutaminase (tTG) and iodinated MGP, respectively, in buffer containing 0.05 M Tris, 2.5 mM Calcium, and 1 mM dithiothreitol (TCD buffer) at 37 degrees. This was incubated for 2 hours. Final concentrations of 0.18 mg/mL FN, 0.18 mg/mL FG, or 0.18 mg/mL VN were incubated with 0.0125 mg/mL tTG and trace amounts of ^125^I-MGP (2.5 ng/mL) containing 30,000 dpm. To determine the effect of antibodies to MGP, 31 *μ*g/mL rabbit anti-MGP IgG was included during incubation (see Figure 2(c)). After incubation, samples were processed immediately for SDS PAGE in sample buffer containing 2% SDS and 2% 2-mercaptoethanol treated at 100 degrees for 2 min. Control incubations omitted the tTG or contained only MGP. The electrophoretic gels were processed by wrapping in plastic film and exposure to Kodak Biomax MS film with HE enhancing screen for the appropriate time at −80°C. After exposure, films were developed as described above. The gels were coomassie blue stained after film exposure to visualize proteins.

DSP crosslinking followed the instructions of the manufacturer. 0.5 mg/mL fibronectin III1-C polypeptide, 0.5 mg/mL FN, 0.5 mg/mL VN, or 0.5 mg/mL ovalbumin were incubated with trace amounts of ^125^-I-MGP (2.5 ng/mL, 30,000 dpm) at ambient temperature for 2 hours in 5 mM phosphate, 75 mM NaCl, 2.5 mM EDTA, and 0.05 mg/mL gelatin pH 7.4. After 2 hours, either 1 mg/mL DSP or an equal volume of DMSO for controls is added at ambient temperature for 30 min. The reaction was quenched for 15 min at ambient temperature with Tris pH 8 to react with free DSP the frozen at −20 degrees for SDS gel electrophoresis or gel filtration chromatography. For SDS-PAGE, samples are diluted with 2% SDS sample buffer pH 6.8 with or without 2% 2-mercaptoethanol and boiled for 2 min. For gel filtration chromatography, samples are diluted with 4 M guanidine HCL, 0.1 M tris, 0.1% tween 20, and 0.5 mg/mL gelatin pH 8.0. Some samples were reduced with 2% 2-mercaptoethanol for 10 min at ambient temperature to break DSP crosslinks before dilution with buffer. Gel filtration chromatography was performed on an AP (Pharmacia-LKB) Sephacryl HR S200 packed in a Bio-Rad column (1.6 × 19 cm) equilibrated with 4 M guanidine, 0.1 M tris, and 0.1% tween 20, pH 8.0. Constant volume fractions (30 drops/fraction) were collected and counted on a Packard Auto-gamma counter to determine the elution position of radioactive MGP. Controls were MGP and ovalbumin incubated with DSP, MGP alone, proteins incubated without DSP crosslinking, and DSP treated samples reduced with mercaptoethanol to reduce and break crosslinks.

### 2.4. Cell Attachment

To determine whether MGP affected cell attachment to fibronectin, HeLa cells were used as an immortal cancer derived cell line that is popular as a model of cell behaviors such as attachment, spreading, and integrin action [34–37]. HeLa cells express integrins that bind to fibronectin and vitronectin. Initial studies demonstrated they had a dose-dependent cell attachment response to increasing concentrations of the matrix proteins. Short term attachment studies within 1 hour after cell release from feeder plates were performed. The short cell attachment duration minimized secretion of HeLa derived attachment proteins and MGP. HeLa cells are grown in Liebovitz L-15 medium supplemented with 10% fetal bovine serum (fbs) with gentamicin and amphotericin B added at the recommended concentrations. 80–95% confluent cells are harvested by release with 5 mM EDTA in calcium-free PBS pH 7.4, washed with 10% fbs L-15 and resuspended to 250,000 cells/mL in serum-free L-15. The cell suspension is used immediately for cell attachment assays below.

In a typical cell attachment assay, 96-well plates are coated with the indicated concentrations of fibronectin in 50 mM sodium bicarbonate buffer pH 9.6 (coat buffer). For control with no fibronectin the well is treated with coat buffer alone. After incubating overnight at 4°C covered with parafilm to prevent evaporation, fibronectin solutions are removed and replaced with the indicated amount of MGP in coat buffer or coat buffer alone as control. After incubating at 37°C in a humidified atmosphere for 3 hours, the second MGP or control solutions are removed and replaced with blocking solution made with 1% BSA in PBS pH 7.4 which has been heat inactivated at 65°C for 30 min. After 1.5 hours at 37°C in a humidified atmosphere, the wells are emptied and 25,000 HeLa cells added in 0.1 mL of serum-free L-15 medium using an 8-well multiwell pipet. Vitronectin mediated cell attachment experiments were performed with the same procedure, except that vitronectin replaces fibronectin in the plate coating. Attachment proceeds at 37°C in a humidified atmosphere for 1 hour. The medium is then gently removed. The wells are immediately washed twice with serum-free L-15 medium and then fixed with 96% ethanol for 10 min and then stained with 0.1% crystal violet dye in distilled water for 30 min at ambient temperature. The dye is removed and the wells washed gently with 0.375 mL of distilled water 3 times. After wells have dried completely, 0.1 mL/well of 0.2% triton X100 is added and incubated at ambient temperature overnight covered with parafilm to prevent evaporation. The plates are read at 595 nm on a Molecular Devices SpectraMax 2500 multiwell plate reader. Data is entered as replicates (usually 6 but a minimum of 4 replicates for each data point) into Prism 2.0 for analysis, nonlinear regression plotting, and statistical analysis. In some experiments the first fibronectin coat is replaced by a first MGP coat and then followed by a second coat of fibronectin to ascertain the effect of switching the order of addition of fibronectin and MGP. In some experiments after an initial coat of fibronectin the remaining nonspecific protein binding sites on the plate are blocked with BSA-PBS before addition of MGP to see if MGP effects required nonspecific protein binding sites on wells. In other experiments the effect of rabbit anti-MGP antibody on MGP enhancement of fibronectin-cell binding was assessed by adding 31 *μ*g/mL (0.0312 A_280_/mL) of anti-MGP IgG in the second coat step containing MGP. An equal concentration of nonspecific rabbit IgG is added as a control.

### 2.5. Cell Spreading

HeLa cells are grown and harvested nonenzymatically as described above and, after washing once with L-15 supplemented with 10% fbs, cells are resuspended in serum-free L-15 medium to 15000 cells/mL. 9000 HeLa cells/0.6 mL are added to each chamber of Lab-Tek 4 chamber glass slides. Lab-Tek chamber slides are previously coated with 0.0, 0.2, or 0.4 *μ*g/mL of fibronectin in coat buffer overnight at 4°C and then a second coat of 3 *μ*g/mL MGP for 3 hours at 37°C and then blocked with heat inactivated BSA-PBS for 1.5 h at 37°C as described above. Cells attach for 2 hours and then are rinsed gently twice with serum-free L-15 medium. Freshly made 1% paraformaldehyde in PBS is added to fix cells for 10 min at ambient temperature, washed PBS for 10 min, and then treated with 0.1% triton X-100 in PBS for 5 min to permeabilize cells. Heat inactivated BSA-PBS is added for 10 min. The BSA-PBS is then replaced with 1 : 40 dilution of Phalloidin Alexafluor488 in heat inactivated BSA-PBS for 20 min. After one wash with heat inactivated BSA-PBS the chambers are washed twice with PBS. The slides are placed in a 10 cm petri dish immersed in PBS and then imaged using 10x or 40x water immersion objectives on a Zeiss Axiophot fluorescence microscope equipped with a digital camera. 16 random microscopic fields are imaged for each condition.

Cell spreading was assessed using the NIH Image software that is publically available. 14–16 random microscopic field images taken on the 10x objective were analyzed, with a minimum of 100 cells analyzed for each condition. The results are presented as the average cell area with standard error. The procedures in NIH Image are as follows: an introduction and tutorial were used to determine cell areas. Briefly, low power images of cells taken with the 10x objective were modified by* autocontrast* and* grayscale conversion* in Photoshop. The images were opened in NIH Image,* scaled* to fit the window, and modified with the* LUTZ* tool to turn cells red then under* Analyze*,* analyze particle*. After examination to manually remove clumped/adherent cells from analysis, the results are copied into excel as area/cell. The data are transferred to Prism for plotting and statistical analysis. The cell spreading analysis is similar to that used in a previous publication measuring cell attachment and spreading on titanium surfaces [38].

### 2.6. Immunolocalization

Paraffin embedded sections of formalin fixed day 19 embryos were obtained as described previously [4, 6]. The sections were deparaffinized with xylene, rehydrated, and boiled for 2 min in 0.01 M sodium citrate, pH 6 for antigen retrieval as described previously [6]. Immunoperoxidase staining followed the Vectastain ABC Elite kit instructions (Vector labs). Sections were blocked with buffer containing normal horse serum at 1.5% then incubated with rabbit anti-MGP at 20 *μ*g/mL or mouse antifibronectin monoclonal antibody (clone IST-3 Sigma) at 1 : 5000 dilution. After an overnight 4°C incubation and washing, biotinylated horse antiuniversal IgG (Vector) is added. After washing, the bound probe is localized by Vectastain ABC peroxidase reagent DAB. Methyl green was used as a counter stain. Controls were nonspecific rabbit IgG at the same concentration or no primary antibody. Images were collected on a Zeiss Axiophot microscope equipped with a PC and 6.3x and 40x objectives.

## 3. Results

### 3.1. MGP Binds to Fibronectin and the First Type III Domain of Fibronectin

**Figure 1:**
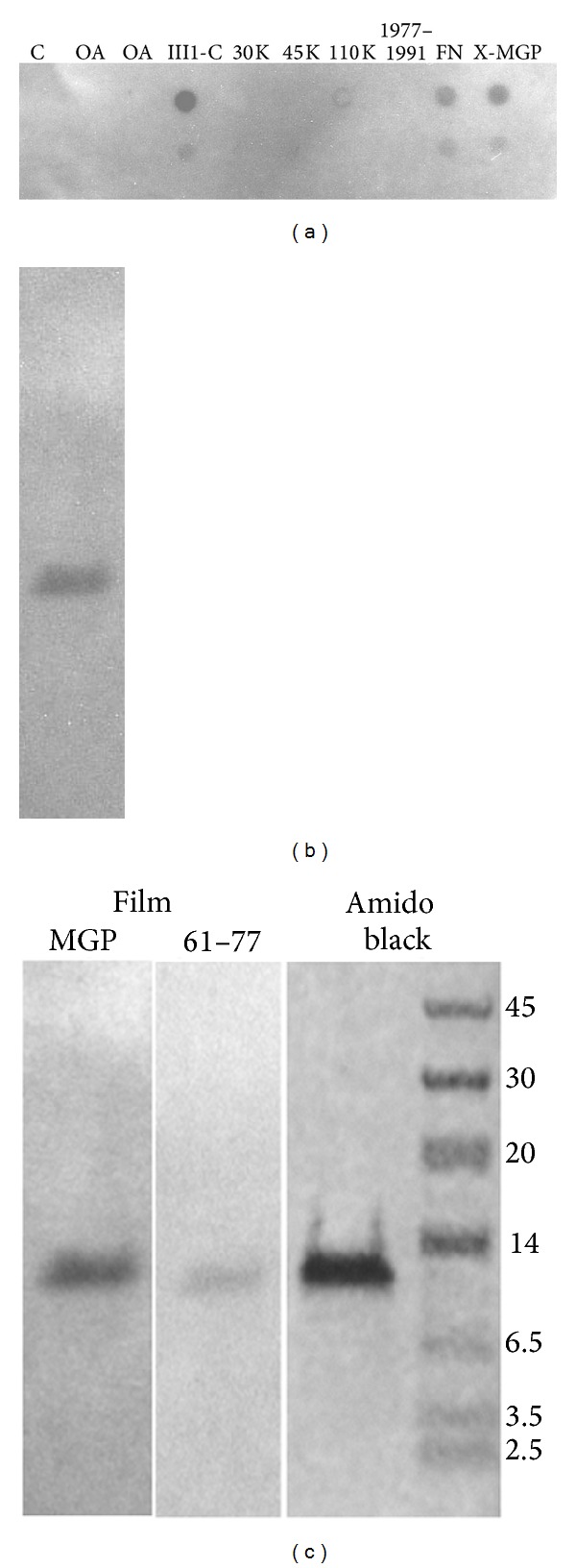
MGP binds to fibronectin and to the first type III repeat domain of fibronectin III1-C. (a) MGP binds fibronectin and fibronectin fragments with type III repeats. Dot blot spots with radioactive MGP probe and 100 microliters of protein solutions were dot blotted onto the nitrocellulose filter, then blocked with 1% BSA-PBS, overlaid with radioactive MGP, washed, and exposed to film as described in methods. C, PBS buffer; OA, 1 mg/mL ovalbumin; FNIII1C, 1 mg/mL fibronectin III1-C; 30 KFN, 1 mg/mL 30 kDA fragment of fibronectin; 45 KFN, 1 mg/mL 45 kDA fragment of fibronectin; 110 KFN, 1 mg/mL 110 kDA fragment of fibronectin; 1991–1997, 1 mg/mL of the cell binding peptide of fibronectin comprised of amino acids 1991 through 1997; FN, 1 mg/mL fibronectin; anti-MGP, 5 *μ*g/mL of anti-MGP IgG from rabbit. The lower row of dot blotted proteins contains 10x lower concentration of proteins. (b) Radioactive MGP probe binds to fibronectin III1-C on far western blot. Fibronectin III1-C is run on SDS-PAGE, electroblotted, blocked and overlaid with iodinated MGP, washed, and exposed to film as described in methods. (c) Both MGP and the 61–77 C-terminal peptide of MGP bind to fibronectin III1-C. Far western blot with either ^125^I-MGP or ^125^I-MGP_61–77_ overlay on fibronectin III1-C and exposed to film as described. Both probes bind to a band that comigrates with a gel band of fibronectin III1-C shown stained with amido black next to molecular weight markers.

As seen in Figure 1(a), overlay of dot-blotted proteins with radioactive MGP demonstrates that MGP binds to fibronectin and fibronectin fragment III1-C (Anastellin). MGP did not bind ovalbumin or other fragments of fibronectin with the exception of the 110 kilodalton fibronectin fragment that weakly binds to MGP. MGP binds to the SDS-PAGE band of fibronectin III1-C (Figure 1(b)). We previously reported that MGP binds to vitronectin and fibronectin but not laminin, type II collagen, osteocalcin, tissue transglutaminase, chondroitin sulfate glycosaminoglycan, biglycan, beta-casein, ovalbumin, or bovine serum albumin [6].

### 3.2. Fibronectin Binds a C-Terminal Peptide of MGP Comprised of Amino Acids 61 to 77

We had previously shown that the MGP_61-77_ peptide binds to vitronectin [6].  Figure 1(c) shows that MGP_61-77_ also binds to the first type III fibronectin domain.

### 3.3. MGP Is Incorporated into Transglutaminase Crosslinked Fibronectin

**Figure 2:**
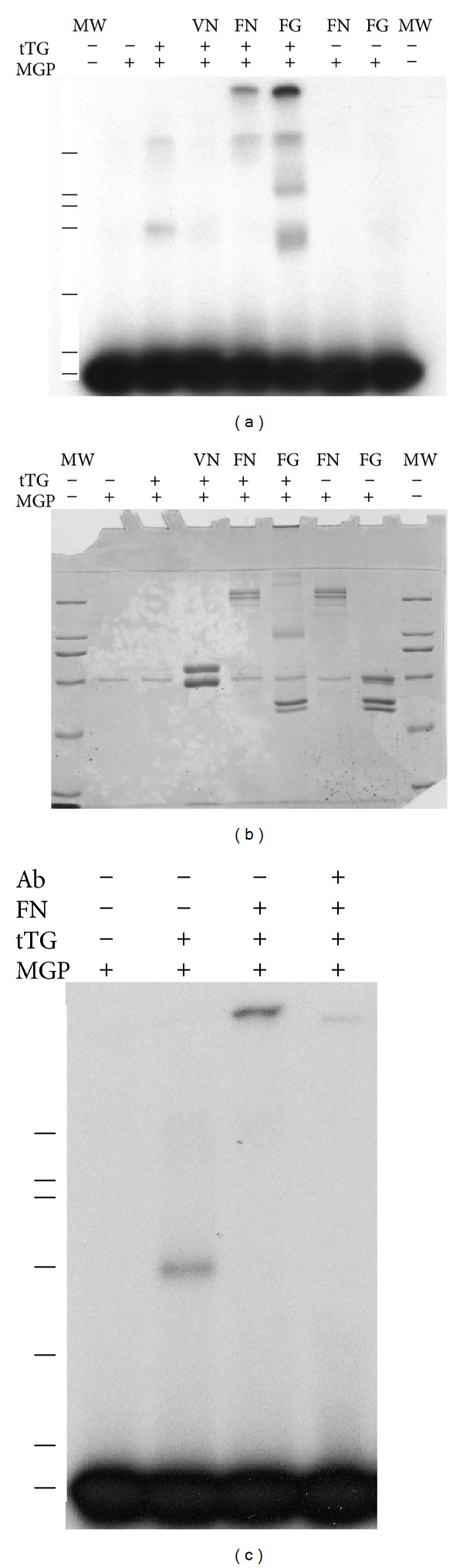
MGP is incorporated into crosslinked fibronectin and fibrinogen by tissue transglutaminase. SDS-PAGE of reaction products of MGP, tTG, and extracellular matrix proteins. Lines indicate 7 marker positions of 200, 116, 97, 66, 43, 31, and 14 kDa. (a) Autoradiograph of 7.5% acrylamide SDS-PAGE gel the legend above indicates whether ^125^I-labeled MGP or tTG was present as + and − symbols; The protein incubated with MGP is shown in the top line, VN, vitronectin, FN, fibronectin, and FG, fibrinogen. MW indicates that molecular weight markers were run in those lanes. (b) Coomassie blue stained 7.5% acrylamide SDS-PAGE gel used for autoradiograph in (a). Molecular weight markers are 200, 116, 97, 66, 43, 31, and 14 kDa. MGP runs with the 14 kDa protein at the dye front on this gel. (c) Autoradiograph of 7.5% SDS-PAGE gel. The furthest right lane marked Ab+ shows inhibitory effect of added anti-MGP IgG on incorporation of MGP into crosslinked fibronectin.

Tissue transglutaminase (tTG) can crosslink fibronectin and fibrinogen to form multimers. MGP incorporated into crosslinked multimers of these proteins. tTG was incubated in mixtures of ^125^I-MGP with fibronectin, fibrinogen, or vitronectin, respectively. The products were analyzed by autoradiographs of samples run on SDS-PAGE to identify shifts in radioactive MGP (Figure 2(a)). Radioiodinated MGP was incorporated into higher fibronectin (FN) and fibrinogen (FG) bands. There was no MGP incorporated into higher molecular weight bands for MGP incubated with FN or FG the absence of tTG. Tissue transglutaminase did not form a high molecular weight band with MGP and vitronectin. Transglutaminase shifted a small amount of MGP to higher molecular weight but not to the size or extent found with either fibrinogen or fibronectin. The band at the bottom of the Figure 2(a) is free radioiodinated MGP.  Figure 2(b) shows the coomassie blue stained gel exposed to film in Figure 2(a). tTG converted only a small fraction of the fibronectin (FN) to multimers as there is little change in lanes with and without tTG. tTG was more efficient at crosslinking fibrinogen (FG) to multimers which can be as additional bands when tTG is present but not in samples without tTG (Figure 2(b)). Note that the coomassie blue stained band in all lanes is BSA, a component of the buffer used to separate radioiodinated MGP from unincorporated iodine during iodination [6]. BSA acts as a free radical scavenger to protect iodinated MGP. MGP in the tTG crosslinked fibronectin lane comigrated at the stacking gel surface or the top of the running gel. The presence of anti-MGP polyclonal antibodies blocked the ability of tissue transglutaminase to incorporate MGP into a fibronectin multimer (Figure 2(c)). Increasing the concentration of tTG increased the amount of MGP shifted to higher molecular weight with fibronectin. Transglutaminase could incorporate MGP into larger complexes of fibronectin but not with vitronectin, ovalbumin, or bovine serum albumin.

### 3.4. MGP Becomes Crosslinked to Fibronectin III1-C

**Figure 3:**
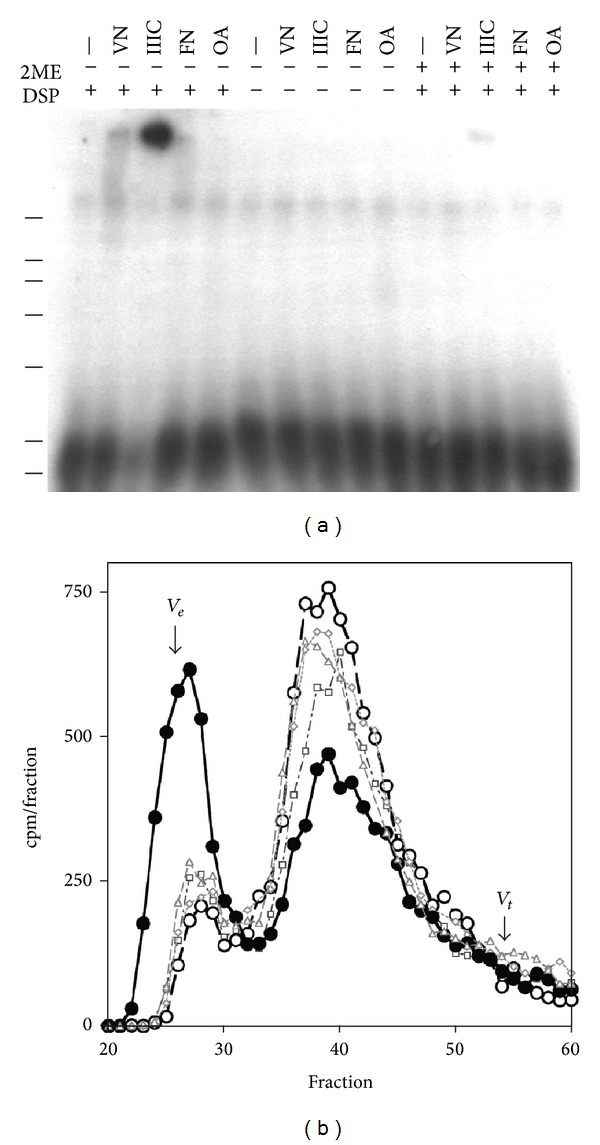
MGP is crosslinked by DSP to vitronectin, fibronectin, and fibronectin III1-C polypeptide. (a) SDS-PAGE of DSP crosslinking reaction products of unlabeled proteins incubated with trace amounts of radioactive MGP. The presence of the crosslinker DSP, reducing agent 2-mercaptoethanol (2ME), is indicated by + or − in the appropriate lane. The protein present in the reaction is indicated above the lane; −, no added protein; VN, vitronectin; IIIC fibronectin III1-C polypeptide; FN, fibronectin; and OA, ovalbumin. (b) Gel filtration column separation of DSP crosslinked MGP and Fibronectin III1-C. Lines indicate 7 marker positions of 200, 116, 97, 66, 43, 31, and 14 kDa. DSP reacted sample containing labeled MGP and unlabeled III1-C (●); DSP reacted solution containing labeled MGP and unlabeled III1-C reduced with mercaptoethanol prior to column loading, (◯); labeled MGP alone (*▵*); Labeled MGP and unlabeled III1-C incubated with DSP vehicle (DMSO) (◊); DSP reacted solution containing labeled MGP alone (□). The exclusion volume *V*_*e*_ was estimated by blue dextran elution, and total volume *V*_*t*_ was estimated by phenol red elution. The column was run with 4 M guanidine containing buffer to disassociate noncovalent interactions (see methods).

Figure 1 showed that MGP and fibronectin III1-C bind on filter overlay assays. Chemical crosslinking could confirm that the MGP and III1-C are in close proximity in solution. ^125^I-MGP and III1-C, vitronectin, or ovalbumin was incubated under associative conditions then reacted with DSP, a disulfide containing homobifunctional crosslinking agent. The prediction is that interacting proteins would become covalently crosslinked in the presence of a crosslinking agent. If DSP is added to a mixture of fibronectin III1-C and ^125^I-MGP, a higher molecular weight crosslinked species is observed on autoradiographs after SDS-PAGE (Figure 3(a), IIIC, −2ME, and +DSP lane). If samples are reduced with 2-mercaptoethanol (2ME), the DSP crosslink is reduced and larger bands disappear (Figure 3(a) IIIC, +2ME, and +DSP lane). No additional higher molecular weight bands are present for IIIC in the absence of DSP crosslinker (IIIC, −2ME, and −DSP lane). The lack of higher molecular weight species is due to disruption of protein interactions in 2% SDS buffer heated to 100°C. MGP and either vitronectin or fibronectin was crosslinked by DSP although not to the same extent as seen with III1-C (Figure 3(a), VN and FN, −2ME, and +DSP lanes). It is possible that the increased crosslinking of III1-C and MGP results from aggregation of the bound complex that increases crosslinking efficiency. Ovalbumin is a negative control for binding; MGP and ovalbumin were not crosslinked by DSP.

If the products of DSP crosslinked ^125^I-MGP and fibronectin III1-C are denatured in 4 M guanidine containing buffer then analyzed by Sephacryl S200 gel filtration, an elution profile with MGP shifted to a peak near the excluded volume, *V*_*e*_, is the result (Figure 3(b), filled circles). If the crosslinked sample is reduced with mercaptoethanol prior to column loading, the elution profile is similar to controls, for example, ^125^I-MGP alone (Figure 3(b), open circles). The ^125^I MGP peak normally elutes around fraction 37 with a minor peak at fraction 28 (Figure 3(b), open triangles). Control reactions with ^125^I-MGP with DSP, ^125^I-MGP + III1-C in the absence of DSP were identical in gel filtration elution profile with MGP alone or the reduced crosslink (open diamonds or squares, resp.). A four-fold decreased amount of III1-C in the mixture with labeled MGP and DSP resulted in a smaller S200 column *V*_*e*_ peak (data not shown). The data show that III1-C and the crosslinking agent were necessary to produce the shift in the MGP peak and are consistent with a close interaction between fibronectin III1-C and MGP.

### 3.5. MGP Enhances Cell Attachment to Fibronectin

**Figure 4:**
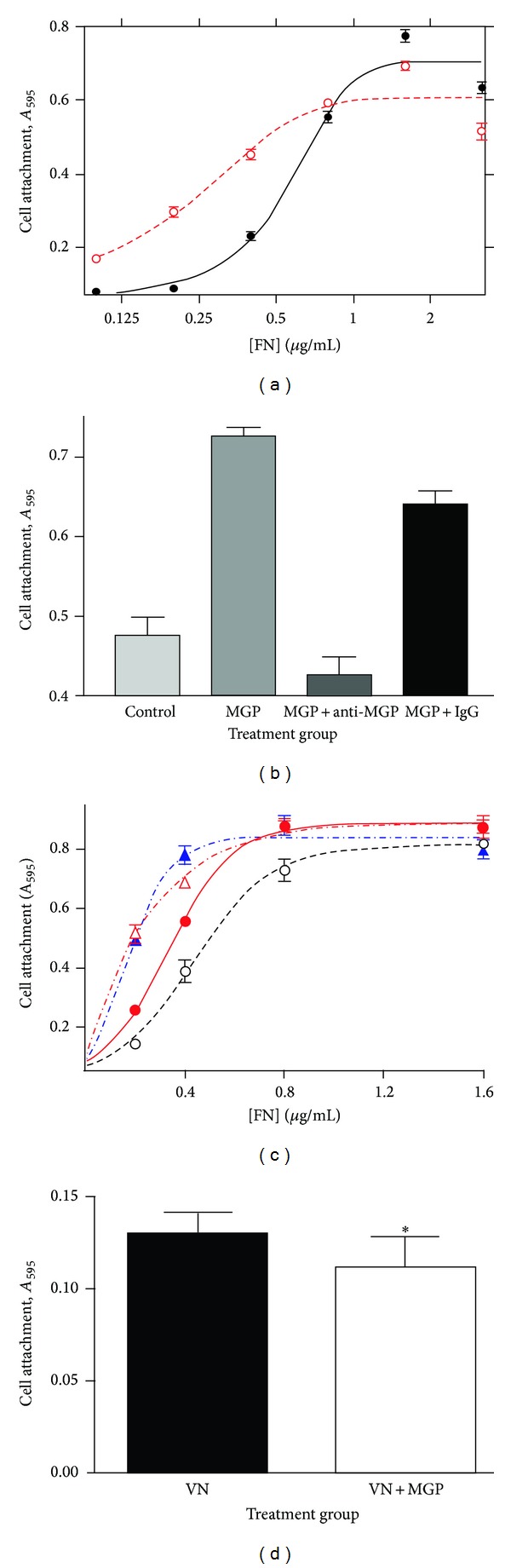
MGP enhances cell attachment to fibronectin. (a) The *y*-axis is HeLa cell binding indicated as absorbance at 595 nm. The *x*-axis is the log of fibronectin concentration from 0 to 3.3 *μ*g/mL either with 3 *μ*g/mL MGP (◯) or control buffer (●). MGP did not enhance cell attachment for 0 fibronectin (not shown, logarithmic plot). (b) Anti-MGP IgG blocks the activity of MGP for enhancing cell attachment to fibronectin. Cell attachment was measured for control, 0.4 *μ*g/mL fibronectin; MGP, 0.4 *μ*g/mL fibronectin plus 3 *μ*g/mL MGP; MGP + anti MGP, *μ*g/mL fibronectin plus 3 *μ*g/mL MGP plus 31 *μ*g/mL anti-MGP IgG; and MGP + IgG, 0.4 *μ*g/mL fibronectin plus *μ*g/mL MGP plus 31 *μ*g/mL rabbit polyclonal IgG. MGP and MGP + IgG were significantly different from control and MGP + antiMGP (*P* ≤ .05). (c) The concentration of MGP enhances cell attachment in a dose responsive manner but does not change maximum cell binding to fibronectin. Experiments were performed as in (a) except that MGP concentrations of 0.0, 0.8, 3, and 6 are compared, and the highest concentration of fibronectin is 1.6 *μ*g/mL. No MGP (◯); 0.8 *μ*g/mL MGP (●); 3 *μ*g/mL MGP (*▵*); and 6 *μ*g/mL MGP (▲). Error bars are SEM for 4–6 replicate binding assays for each data point. Lines are nonlinear regression lines derived by Prism software. (d) MGP inhibits cell binding to vitronectin. VN, 0.2 *μ*g/mL vitronectin; VN + MGP, 0.2 *μ*g/mL vitronectin plus 0.8 *μ*g/mL MGP. Error Bars are SEM for 8 replicate binding assays. The ∗ indicates significant difference (*P* < .05).

Cells attach to fibronectin in a dose dependent manner.  Figure 4(a) shows that cell attachment increases with increasing fibronectin until a maximum is reached at fibronectin coating concentrations between 0.8 and 3.2 *μ*g/mL (solid line with closed circles). MGP enhanced cell attachment to fibronectin (open circles with the dashed line, Figure 4(a)). The cell attachment curve for equal amounts of fibronectin is shifted to the left if MGP is added at 3 *μ*g/mL. The result is analogous to increasing the apparent affinity of cells for equivalent amounts of fibronectin (Figure 4(a)). Maximum cell binding occurred near 0.8 *μ*g/mL fibronectin. MGP alone did not mediate cell attachment. In addition, MGP did not increase cell attachment beyond the maximum observed for fibronectin alone. The lack of inherent cell attachment activity for MGP is in agreement with the absence of cell binding activity previously reported [39]. The results prove that MGP augments cell attachment to fibronectin.

In contrast to MGP augmenting cell attachment to fibronectin, MGP did not augment attachment of cells to vitronectin.  Figure 4(d) shows that MGP inhibits cell attachment to vitronectin. There was a small inhibitory effect of MGP on cell binding to vitronectin, but in some experiments there was no significant effect. Under the same conditions and cell line, MGP consistently augments cell attachment on fibronectin but not on vitronectin.

Antibody to MGP inhibited the ability of MGP to augment cell attachment (Figure 4(b)). Anti-MGP rabbit IgG abolished the ability of MGP to enhance cell attachment to fibronectin (MGP compared to MGP + antiMGP, *P* ≤ .05), but an equal amount of nonspecific rabbit IgG did not. The presence of IgG at the concentrations used did not affect cell attachment to fibronectin, because anti-MGP IgG or control IgG added in the absence of MGP was identical to the control fibronectin alone (Control, Figure 4(b)).

Increasing amounts of MGP dose dependently enhanced cell attachment to fibronectin (Figure 4(c)). MGP affects the apparent affinity of cells for fibronectin not the maximum binding. A dose response curve of increasing MGP (0.0, 0.8, 3.0, and 6.0 *μ*g/mL) reveals a change in apparent affinity with an increasing shift of the binding curve to the left indicating increased cell attachment at each concentration of fibronectin. Again, maximum binding of cells to fibronectin was not affected (Figure 4(c)). There was no inherent MGP cell attachment activity, because cells did not attach even at the highest amounts of MGP (6.0 *μ*g/mL) if fibronectin was not present.

### 3.6. Cell Spreading on Fibronectin is Augmented by MGP

**Figure 5:**
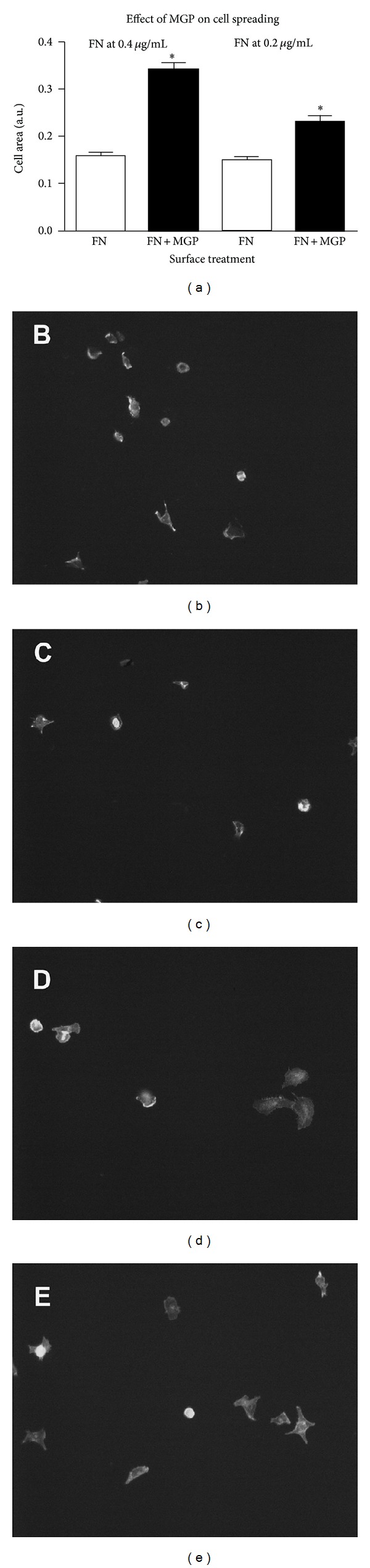
MGP augments cell spreading on fibronectin. In Panel (a) the graph shows the calculated cell area/cell for fibronectin coated surfaces compared to fibronectin plus MGP coated surfaces. FN is fibronectin alone open bars at 0.4 *μ*g/mL (left) or 0.2 *μ*g/mL (right); FN + MGP dark bars is the indicated concentration of fibronectin plus MGP at 3 *μ*g/mL. The asterisk indicates that the combination of FN + MGP was significantly different from FN at each concentration of FN (*P* ≤ .0001, error bars are SEM). The average cell area from a minimum of 100 cells in 14 to 16 random microscopic fields is shown. Cells were allowed to attach for 2 hours in serum-free medium then fixed, stained, and imaged and the cell area quantified. Area per cell was determined by NIH Image software as described in experimental procedures. Panels (b) and (c) are images of cells attached to fibronectin alone (0.4 *μ*g/mL), and Panels (d) and (e) are images of cells attached to fibronectin plus MGP (0.4 *μ*g/mL and 3 *μ*g/mL, resp.).

Cells attach and spread on fibronectin as integrins and other cell adhesive proteins form focal adhesions which connect to the actin cytoskeleton. To determine whether MGP affects cell spreading on fibronectin, the average area of cells attached to fibronectin alone or fibronectin plus MGP was determined by calculation of average cell area from over 100 cells as described in experimental procedures. MGP induced significantly greater cell spreading at each fibronectin concentration (Figure 5(a), FN versus FN + MGP, *P* ≤ .0001 indicated by asterisk). There were too few cells attached in the MGP alone wells to measure spreading (MGP does not have cell adhesive activity on its own).

The appearance of selected microscopic fields of cells adherent on 0.4 *μ*g/mL fibronectin coated surfaces is shown is in Figures 5(b) and 5(c). The appearance of selected microscopic fields of cells on the same concentration of fibronectin plus MGP is shown in Figures 5(d) and 5(e). The enhanced spreading of the cells with fibronectin and MGP is visible.

### 3.7. MGP Localizes in Embryonic Tissues in the Same Areas as Fibronectin

**Figure 6:**
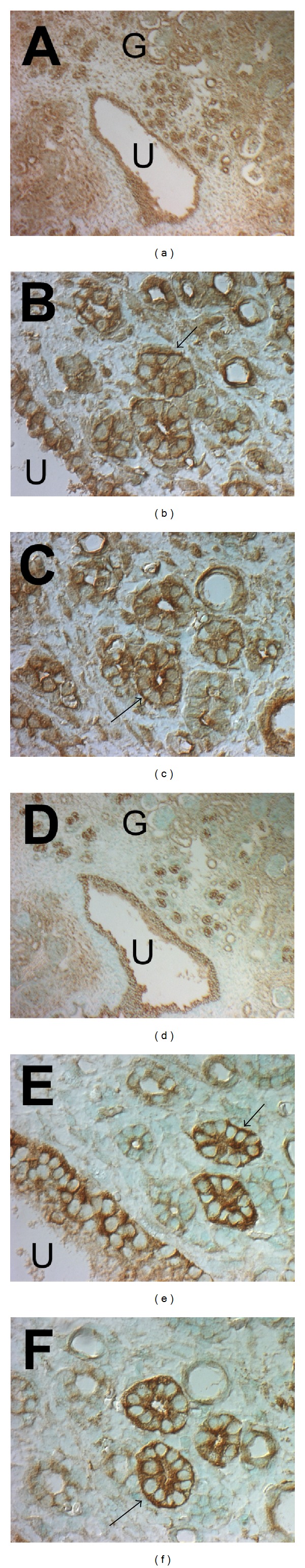
MGP and fibronectin localization in the embryonic rat kidney. (a)–(c) serial section of E20 rat kidney in which fibronectin is localized with mouse-anti-fibronectin and Vectastain ABC elite universal peroxidase-diaminobenzidine and counterstained with methyl green. (d)–(f) Serial section of E20 rat kidney in which MGP localized with rabbit-anti-MGP, Vectastain ABC elite universal peroxidase diaminobenzidine and counterstained with methyl green. A and D are low power images of the same region of kidney taken with the 6.3x objective. U is the lumen of the developing ureter, and G is a developing glomerulus. Fibronectin localized in B and C taken with a 40x oil immersion objective. U is the lumen of the developing ureter. Arrows indicate tubules identical to those shown in the serial section. MGP localized in E and F, image of equivalent fields as B and C, taken at the same magnification with a 40x objective. U is the developing ureter. Arrows indicate intensely stained tubules for comparison.

It was previously shown that MGP in the rat embryonic kidney localizes in the developing ureter and the collecting tubules [6]. Fibronectin is widely distributed in the rat embryonic kidney, including in the ureter and collecting tubules (Figures 6(a), 6(b), and 6(c)). Localization of MGP in a serial section reveals that fibronectin is present in some of the same collecting tubules and ureter epithelium as MGP (Figures 6(d), 6(e), and 6(f)). Arrows in Figures 6(b), 6(c), 6(e), and 6(f) indicate similar structures in serial sections with both fibronectin and MGP. Fibronectin has a wider distribution within the developing kidney, being present in other tubules with little or no MGP, in connective tissue areas, and in forming glomeruli (Figures 6(a)–6(c)). Developing glomeruli has little or no MGP at this stage of development (Figure 6(d)).

## 4. Discussion

MGP binds fibronectin. This confirms the results from a previous publication that showed that MGP binds to fibronectin, vitronectin, and fibrinogen but not tissue transglutaminase, type II collagen, fibromodulin, osteocalcin, osteonectin, heparin, decorin, casein, or ovalbumin [6].

The current study shows that MGP binds to a region of fibronectin made up by its first type III repeat called III1-C or anastellin [40]. MGP did not bind the 30, 45 kilodalton fragments of fibronectin that do not contain a type III repeat [40, 41]. MGP showed some affinity for a 110 kilodalton fragment of fibronectin that is comprised of many type III repeats [40, 41].

A region of MGP comprised of amino acids 61–77 was proposed as an extracellular matrix binding region [6]. The current study shows that MGP_61–77_ peptide binds to fibronectin and also to the fibronectin III1-C fragment. All MGPs isolated from mineralized tissues have been truncated at the C-terminus. For example, human MGP is originally 84 amino acids long but has 7 amino acids removed from the C-terminus, so that the mature protein is 77 amino acids long and ends with the peptide sequence identical to the MGP_61–77_ peptide used for this study [42]. While bovine MGP was used in this study, the results imply that the human MGP will bind to extracellular matrix proteins via a sequence at its carboxyl terminal. The proteolytic processing of the MGP C-terminal to remove amino acids 78–84 may relate to activating a protein interaction site [42].

Fibronectin is a substrate for crosslinking by tissue transglutaminase [43]. The crosslinked multimerized fibronectin formed by incubation with tTG is analogous to the insoluble matrix fibronectin present in many tissues. The close association of MGP and fibronectin suggested that MGP may become incorporated into fibronectin crosslinked multimers. Indeed, addition of MGP during crosslinking of fibronectin by tTG resulted in its incorporation into a larger multimer form of fibronectin. The ^125^I MGP remained with the fibronectin multimer after boiling in 2% SDS that dissociates the MGP and fibronectin interaction. Thus MGP more strongly associated with crosslinked fibronectin than with fibronectin. It is possible that transglutaminase crosslinks MGP to fibronectin, although further studies are necessary to prove crosslinking occurs and determine which amino acids of MGP and fibronectin are involved.

The shift of smaller amounts of MGP to higher molecular weight by tTG suggested it may become crosslinked to itself or to tTG, but we were unable to directly establish that MGP is a substrate of tTG. To prove MGP was a substrate for tTG we attempted incorporation of radiolabeled putrescine as described [44]. The results were inconclusive, with minimal incorporation of radiolabel into MGP. Previously we had shown that MGP does not bind tTG [6]. MGP may require a coreactant like fibronectin or fibrinogen to become efficiently crosslinked. The association of MGP with crosslinked fibronectin confirms a close association of MGP with fibronectin. MGP also becomes incorporated into tTG crosslinked fibrinogen. MGP was previously shown to bind to fibrinogen [6]. Although MGP binds to vitronectin, tTG did not incorporate MGP into a high molecular weight component with vitronectin. The results suggest that MGP may become a component of crosslinked fibronectin and fibrinogen in vivo.

The fibronectin III1-C domain is a binding site for MGP. This has been established by overlay assays and by DSP crosslinking of MGP to the fibronectin III1-C polypeptide. Overlay assays show that the C-terminal MGP 61–77 amino acid peptide can bind to fibronectin and to the III1-C polypeptide. MGP used in these studies is isolated from calcified bone and is gamma carboxylated. A noncarboxylated form of MGP (ucMGP or Glu-MGP) accumulates in calcified atherosclerotic plaque [22, 30]. Further studies will be necessary to determine whether Glu-MGP and MGP share binding for fibronectin and the III1-C domain.

MGP enhances cell attachment to fibronectin. This is not simply an additive effect of providing more adhesive protein, because MGP by itself has no adhesive activity. The present results support a report on lack of adhesive function for MGP [39]. The lack of inherent cell adhesion activity suggests that MGP acts by altering the ability of cells to bind fibronectin. MGP binding of fibronectin seems to be the key, because the enhanced cell attachment was observed in BSA-coated surfaces in which MGP-fibronectin binding was the only way that the enhancement could occur. MGP binding of a protein was not in itself sufficient to enhance cell attachment. MGP enhanced cell attachment to fibronectin but not to vitronectin, another extracellular matrix protein bound by MGP [6]. MGP also enhanced spreading of cells on fibronectin.

These studies explored the mechanism of MGP binding to fibronectin and the effect of exogenous fibronectin and MGP on cell attachment and spreading. HeLa cells are a well characterized cell line that is a popular model for cell attachment. HeLa cells express the integrins for fibronectin and vitronectin binding [34–37]. The goal was to characterize the effect of MGP for fibronectin binding and cell-fibronectin interactions in a short, 1 hour period when cellular synthesis and secretion of fibronectin, MGP, and other attachment proteins could be minimized.

The literature indicates that the effect of MGP may be tumor type dependent. There has been a positive correlation of MGP expression with tumor progression and poor prognosis in glioma [15, 16, 45], but a negative correlation of MGP expression with tumor progression and metastasis in renal and prostate carcinoma [13] and decreased MGP has been found in colon carcinoma [46]. MGP enhances glioma cell migration [16]. MGP promotes angiogenesis and growth in xenografts of glioblastoma [17]. Future studies should determine whether MGP has the effect in multiple normal and neoplastic cell types. Other studies could determine the effect of MGP for migration and invasion assays in HeLa compared to other cancer cell types, but longer term studies need to consider the ability of cells to synthesize fibronectin, MGP, integrins, or metalloproteinases that affect migration and invasion.

The expression of MGP mRNA is very high in the embryonic rat kidney [1, 4]. We demonstrated that MGP protein is also synthesized during this time and that localization of MGP mRNA was strongest in the developing ureter and collecting tubules on embryonic days 17–19 [4]. MGP protein localized to rat embryonic kidney ureters and to developing collecting tubules [6].

Immunolocalization studies showed MGP localized in embryonic ureters and collecting tubules also contained abundant fibronectin. However, fibronectin is also enriched in areas that contain little or no MGP. Therefore MGP was not deposited in all fibronectin containing matrices. The localization supports the possibility of interaction between MGP and fibronectin. However, further studies are needed to prove that the interaction occurs in vivo.

Calcification inhibition and BMP-2 binding requires posttranslational carboxylation to produce GLA in a vitamin K-dependent step [22, 26, 29]. The phosphorylation of 3 serine residues at the amino terminal end of MGP is necessary for its secretion to inhibit deposition of hydroxyapatite in the extracellular matrix [2, 47]. The amino terminal 1–54 amino acids of MGP contains the phosphoserines and GLA necessary for calcification inhibition, whereas a C-terminal peptide MGP_61–77_ has been shown previously to bind vitronectin and in the current studies to bind fibronectin [6, 26].

It is not clear whether the binding of fibronectin or modulating the cell response to fibronectin is related to the calcification inhibitory activity of MGP. It is more likely that this MGP property is related to migration-promoting activity that is demonstrated for glioblastoma cells [16]. It is possible that MGP is a multifunctional protein that can act to modify cell-matrix interactions during embryonic life or in cancer cells and also as a calcification inhibitor in selected adult tissues. This could help explain why many tissues that contain MGP synthesizing cells do not become calcified in MGP knockout animals and in Keutel syndrome, a human disease characterized by loss of MGP function.

## 5. Conclusions

MGP binds to fibronectin. MGP binds via a 61–77 amino acid sequence present at the physiologic C-terminus. Fibronectin binds to MGP via its first type III domain called anastellin or III1-C. MGP can become part of transglutaminase crosslinked multimers of fibronectin, suggesting it may be a component of fibronectin matrices. Cells attach and spread better on fibronectin and MGP coated surfaces than to fibronectin alone, even though MGP itself has no cell attachment activity. In tissues, MGP localizes near fibronectin, suggesting that interactions between the proteins are possible in vivo. The ability of MGP to alter cell interactions with fibronectin is a potential reason for cancer cells and certain embryonic cells to overexpress MGP.

## Abbreviations

MGP: Matrix Gla protein
SDS: Sodium dodecyl sulfate
PAGE: Polyacrylamide gel electrophoresis
kDA: Kilodaltons.
